# A Taxonomy for Health Information Systems

**DOI:** 10.2196/47682

**Published:** 2024-05-31

**Authors:** Anna Janssen, Candice Donnelly, Tim Shaw

**Affiliations:** 1 Faculty of Medicine and Health The University of Sydney Sydney Australia

**Keywords:** eHealth, digital health, electronic health data, data revolution, actionable data, mobile phone

## Abstract

The health sector is highly digitized, which is enabling the collection of vast quantities of electronic data about health and well-being. These data are collected by a diverse array of information and communication technologies, including systems used by health care organizations, consumer and community sources such as information collected on the web, and passively collected data from technologies such as wearables and devices. Understanding the breadth of IT that collect these data and how it can be actioned is a challenge for the significant portion of the digital health workforce that interact with health data as part of their duties but are not for informatics experts. This viewpoint aims to present a taxonomy categorizing common information and communication technologies that collect electronic data. An initial classification of key information systems collecting electronic health data was undertaken via a rapid review of the literature. Subsequently, a purposeful search of the scholarly and gray literature was undertaken to extract key information about the systems within each category to generate definitions of the systems and describe the strengths and limitations of these systems.

## Background

The collection and use of electronic health data (EHD) is common in contemporary society due to the high level of digitization. As the amount of electronic data continually increases, many sectors refer to the phenomenon as a data revolution [1]. The health sector mirrors this trend and is increasingly digitized, particularly in response to the COVID-19 pandemic [2,3]. Information and communication technologies (ICTs) that are enabling EHD collection include, but are not limited to, electronic health records (EHRs), patient administrative systems, web-based information from social media and other sources, data collected within apps, and data from wearable devices [4].

The widespread use of these ICTs enables the development of a rich health data ecosystem, which has great potential to enhance existing approaches to patient self-management, service delivery, and new care models. Different types of EHD and their potential have been investigated in several contexts. This includes how EHD is used to improve health and well-being [5,6], manage chronic conditions [7,8], identify and respond to public health concerns [9], and support quality improvement activities [10-12].

Despite the large amount of EHD collected, it continues to be underused by many health care organizations and providers [13]. There is a gap in understanding what constitutes actionable data. Actionable data have been described as data that enable the user to make an informed decision to solve a specific problem [14,15]. In the health sector, actionable data have been described as data that can improve the quality, outcomes, or cost of care [16]. Another challenge in using EHD is the increasingly large amount of data held by private industry, as the organizations that develop the technology often retain custody of its data [17,18].

To transform EHD into actionable data, unique challenges need to be overcome. One of the most widely acknowledged challenges is adequate infrastructure for EHD use. Infrastructure challenges include (1) the inability to access qualified and experienced technical experts for extracting and analyzing data and (2) deeply ingrained interoperability issues between existing data sources [19]. A further challenge is that EHD is incredibly complicated and fragmented within individual departments and health care organizations [19]. Data are often not centralized within organizations, and a cultural change is required [20,21]. Extracting data from these silos brings new challenges related to privacy and the safety of data transmission within a connected health system [16]. Finally, many health care organizations only have small data, which have significant potential for real-time analytics and presentation in digestible formats, that is, dashboards [13]. However, much of the literature has focused on big data use [22,23], with limited research or understanding of the value of small health data sets for generating meaningful insights.

There are many benefits to increasing the actionability of health data. It can provide a foundation for learning health systems. Such systems transform routinely collected EHD into useful information to improve health care quality and outcomes and support timely decision-making [24]. Another benefit is the increased accessibility and value of EHD for health professionals and organizations. It is particularly important that health professionals see value from their data input. Since the implementation of EHRs, health professionals have been increasingly required to take an active role in data entry. Such data entry has been repeatedly noted as adding a significant workload burden [25]. Although there is a workload burden, it is also postulated that health professional–collected data will be of higher quality than other sources [26]. Demonstrating the benefits of EHD to health professionals will also likely increase their buy-in to data collection, which is essential in improving the completeness and quality of EHD. Expanding the utility of EHD would also benefit consumers in supporting new care models such as virtual care [27]. Furthermore, actionable and transparent EHD can support personalized and patient-centered care [28].

**Figure 1:**
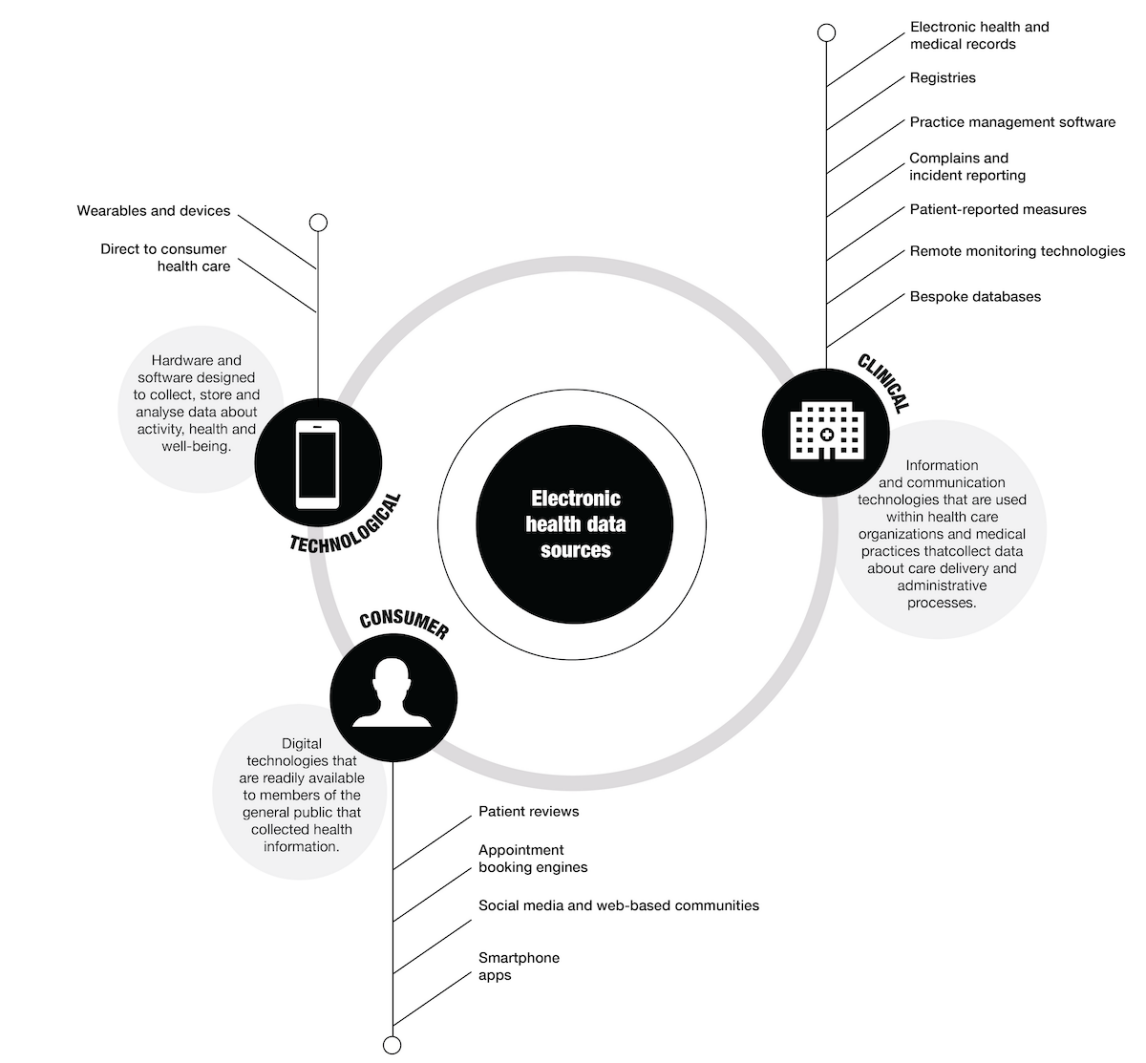
A visualization of the different health information systems that collected electronic health data and how they can be broadly categorized.

This paper aims to present a classification of health information systems used in health care and describe how the information collected by these systems contributes to the health data ecosystem. Furthermore, the paper aims to better explore what constitutes actionable data in health care and contextualize the role of different data sources for this purpose. A key contribution of this study is the development of a health information system taxonomy to help classify the breadth of data collected about health (Table 1) and a visualization of how the different ICTs that collect EHD can be categorized (Figure 1).

This paper addresses a gap in the current literature by providing an overarching description of the health data ecosystem. Recent research has focused on describing certain types of EHD, that is, imaging, administrative, and genomic data [29], or classifying subcategories of health information systems, particularly clinical information systems [30,31].

**Table 1:** Overview of the different health information systems, the data they collect, and the examples of how the data have been actioned.

Health information system		Description of the system	Actionability of data			
**Clinical data sources**						
	EHRs^a^ and EMRs^b^	Repositories of patient health information created by health professionals		Reducing errors as a single source of truth for health information Improving governance, organizational processes, or health service delivery Supporting research, quality improvement, and reflective practice Providing data for clinical decision support systems and AI^c^ technologies		
	Registries	Information systems that collect uniform data to evaluate outcomes for a specific population, disease, condition, or exposure		Supporting disease management Real-time source of information for monitoring disease in the community Understanding population health trends Supporting research, quality improvement, and reflective practice		
	Practice management software	Software designed to manage the everyday activities of medical practices	Reducing errors as a single source of truth for health information Improving workflow efficiency Underpinning clinical decision support systems			
	Consumer complaints and incident reports	Repositories of data patients report about problems they experience when interacting with a health care organization	Supporting research, quality improvement, and reflective practice Improving governance, organizational processes, or health service delivery			
	Hospital administrative information systems	Platforms that collect data about administrative information and billing information within health care organizations	Supporting research, quality improvement, and reflective practice Monitoring adverse events Improving efficiency of health service delivery			
	Patient-reported outcome measures	Questionnaires that measure patients’ perceptions of a disease or its treatment on their health	Identifying patient perspectives on issues important to them about their health and health care Supporting research, quality improvement, and reflective practice Improving governance and organizational processes			
	Diagnostic information systems	Information systems that collate and report on results from different diagnostic processes including blood tests, radiology, and imaging	Supporting research, quality improvement, and reflective practice Providing data for clinical decision support systems and AI technologies			
	Electronic prescribing systems	Digital technologies for managing collection, distribution and storage of scripts	Can improve the efficiencies of prescribing processes			
	Remote monitoring platforms	Digital technologies that can passively monitor, assess, and potentially manage decisions about care	Data are collected continuously about wearers’ health Lack of guidelines or tools to inform best practice use of data by health professionals			
	Bespoke databases	A variety of platforms exist, including repositories of information created by members of the health workforce to collect data that they prioritize and health information collected by medical devices within health care organizations	Supporting research, quality improvement, and reflective practice Adapting to the priorities and data collection needs of individual health professions, specialty groups, or organizations			
**Consumer and community data sources**						
	WPRs^d^	Information consumers publish on review websites on the internet, sharing reviews of clinical encounters with health professionals or health care organizations	Identifying patient perspectives of issues important to them about their health and health care Supporting information-seeking behaviors Improving governance, organizational processes, or health service delivery			
	Appointment booking systems	Web-based applications that enable consumers to book appointments with health professionals and manage those bookings	Reducing health care organization costs Improving patient-centeredness in health care and health service delivery			
	Web-based communities	Information shared by individuals on the web via social media and virtual forums, or input into web-based search engines, can be used to understand community health and well-being	Supporting health communication Improving consumer knowledge about their health Identifying patient perspectives on issues important to them about their health and health care			
	Web-based search engines	Data collected by interacting with web-based search engines.	Understanding population health trends A real-time source of information for monitoring disease in the community			
	Smartphone, web, and desktop apps	Self-contained programs that run on the internet, on smartphones, or on computer operating systems that are designed to improve health and well-being	Improving the completeness and quality of EHD^e^ collection Support disease management			
**Technology-collected data sources**						
	Wearables and devices	Computer hardware that an individual wears as an accessory or by attaching to their clothing, which passively collects data about their activity	Supporting disease management Enabling new, more personalized models of care Collecting information on individual health and well-being behaviors			
	Direct-to-consumer health care	A range of emerging products and services that provide technology at the first point of care and may use AI to triage patients	A large amount of data are being collected by these systems To date, access to these data have largely been controlled by product vendors			

^a^EHR: electronic health record.

^b^EMR: electronic medical record.

^c^AI: artificial intelligence.

^d^WPR: web-based patient review.

^e^EHD: electronic health data.

## Developing the Taxonomy

The EHD ecosystem underpins much of digital health, but it is complex and difficult to navigate. To tackle this issue, the authors have created a comprehensive taxonomy that categorizes essential health information systems. This taxonomy serves as a valuable resource for the numerous individuals in the digital health workforce who may lack extensive informatics expertise but are bound to encounter such systems in their roles. These individuals in the digital health workforce may regularly *interact* with health data and use actionable insights from the data to inform aspects of their job, or they may be interacting with computer scientists, data analysts, informaticians, or other data analysts regularly [32]. The taxonomy is for these individuals who need a nontechnical overview of key systems and the reasons the systems were designed to understand the strengths and limitations of the data they collect.

A taxonomy is an approach for specifying the characteristics of objects and categorizing them to help understand complex domains [33]. A wide range of methodologies can be used for developing taxonomies, and the literature indicates that inductive, deductive, and intuitive approaches can all be used to good effect [34]. Methodologies for developing taxonomies vary across disciplines. The inherently multidisciplinary nature of digital health means there is not a single, widely recognized methodology for developing taxonomies in this space. The taxonomy *development* described in this paper was guided by a modified version of design science research and an improved method for taxonomy development [33]. This was done by applying the following steps: (1) problem definition, (2) definition of objectives for the taxonomy, (3) specifying end conditions, and (4) design and development.

The problem definition (step 1) and objectives for the taxonomy (step 2) are outlined in earlier sections of this paper. The end conditions for the taxonomy (step 3) were chosen to be the point of adequate conciseness [34], that is, the point at which the taxonomy had become meaningful without becoming unwieldy or overwhelming to end users. Finally, the design and development process for the taxonomy (step 4) was undertaken via a rapid review of existing taxonomies or classifications of EHD systems. This review was used to create an initial categorization for the objects in the taxonomy, the key information systems for inclusion in the taxonomy, and the higher-level categorizations for these systems. This initial categorization was discussed by the researchers (AJ and CD) and augmented with missing systems. A purposeful search of the scholarly and gray literature was subsequently undertaken to extract key information about the EHD systems within each category, generate definitions, and describe the strengths and limitations of these systems.

A limitation of this taxonomy is that it did not undergo an evaluation process. However, it has been noted in the literature that this is a common limitation of taxonomies, which frequently go through iterative development but do not have a final evaluation stage [33].

## Health Information Systems

### Summary of EHD Sources

EHD can be broadly classified into 3 categories, described in depth in subsequent sections of this study. Refer to Table 1 for the taxonomy of key health information systems. Figure 1 visualizes the higher-level categorizations of these systems. The three categories are as follows:

1. Clinical data sources: digital technologies that are used by health care organizations to collect EHD. It includes technologies such as electronic health and medical records, registries, practice management software, patient-reported measures, and bespoke databases collecting information from various medical devices used in the clinical setting.
2. Consumer and community data sources: consumer technologies that collect data about the health and well-being of the individuals using the technology. It includes technologies such as desktop and smartphone apps, social media, and web-based communities.
3. Technology-collected data sources: systems that passively collect data about people with digital technology. It largely describes data collected by wearables and other devices.

### Clinical Information Systems

#### Overview

There are a variety of definitions of clinical data. At the highest level, clinical data can be defined as data collected during the care delivery process of health care organizations [35]. Although clinical data mostly refers to data about patients, the data are used for a wide range of applications beyond the delivery of care, including billing [36] and research [37]. The systems that collect clinical data are typically those used by organizations to deliver health services or to monitor processes and outcomes of care. Data in this category are commonly entered by a member of the health workforce. However, some systems may also passively collect metrics on end-user interactions with the technology.

#### Electronic Health and Medical Records

The terms EHR and electronic medical record (EMR) are frequently used interchangeably, but there are subtle differences between the 2 technologies. EMRs are repositories of patient health information created by health professionals to capture data related to specific clinical encounters [38]. EHRs include EMR functions but also have additional ones. The crucial difference is that EHRs are designed to share information across different health care settings and potentially between health professionals and patients [39]. Typically, EHRs are designed to collect both structured and unstructured data. Data collected by EHRs can be beneficial in many contexts, including at the clinical, organizational, and societal levels [40]. Clinical benefits include a reduction in medical errors and improving the completeness and accuracy of data [41]. EHR and EMR data have also been useful for supporting quality improvement activities such as audits and feedback [42] and can potentially enable workplace learning and reflective practice [43].

#### Registries

Clinical registries are information systems that collect uniform data to evaluate specified outcomes for a population, disease, condition, or exposure [44]. Registries can be implemented for a range of purposes, including quality improvement [37], disease monitoring [10], device surveillance, and health care services monitoring [10]. Registries are generally designed to collect structured data. Health professionals usually input data retrospectively using strict definitions aligned with informatics infrastructure [45]. However, there is growing interest in the use of e-registries, which use ICTs to enable systematic, automated, and longitudinal collection, retrieval, and analysis of data [46].

#### Practice Management Software

Practice management software is designed to manage the everyday activities of a medical practice [47]. Historically, practice management software was largely focused on supporting billing [48]. Contemporary systems often incorporate a range of administrative functions such as patient scheduling and registrations, financial reporting, and collections management as well as elements such as patient records and potentially patient communications functionality [48]. Practice management software usually collects a combination of structured and unstructured data. Some practice management software has started incorporating more sophisticated tools for supporting clinical care, such as tracking vaccinations and recording test results.

#### Consumer Complaints and Incident Reports

Consumer complaints and incident data can include problems as diverse as organization parking issues to physical harms and sentinel events [49]. These data can be captured on a range of different platforms. As such, there is variation in the data collected across organizations, which can influence what data points are captured. Generally, the data collected by these systems are unstructured.

#### Hospital Administrative Information Systems

Health care organizations use a variety of platforms to collect data about administrative aspects of care and clinical billing [36]. One of the most common types are patient administration systems (PASs), which capture a range of data, including demographic information about patients and interactions between the health care organization and the patient [50]. These administrative data systems indirectly capture information about care delivered, but it is not their primary purpose [51]. PAS data are often carefully structured, particularly data related to payments, as they may need to be provided by health care organizations with different PAS to health insurance companies with different processing software [52]. This is done by coding PAS and other data collected within a health care organization and providing the coded data to payers, commonly called claims data, for reimbursement purposes [53].

#### Patient-Reported Outcome Measures

Patient-reported outcome measures are data collected using questionnaires that measure patients’ perceptions of disease management and outcomes [54]. They do not have to be collected electronically, but they often are. These data are particularly valuable for assessing the patient-centeredness of care and how effectively care responds to patient needs [55]. They can be reported as individual data or in an aggregated form [56]. These types of information systems are increasingly designed to collect structured data, but this can vary depending on the design of the individual data collection tool.

#### Diagnostic Information Systems

These systems are designed to collect data generated by diagnostic results, including imaging, radiology, and pathology test results, and to report genomic data to inform diagnosis and guide therapeutic approaches [57]. Data from these systems are often integrated into EHR, EMR, or PAS within health care organizations and practice management software in primary care in addition to being stored in independent information systems [21]. Radiology information systems and their equivalents collect reports on test results for use by the health workforce [58].

#### Electronic Prescribing

Electronic prescribing systems are used by pharmacies to generate digital prescriptions instead of paper-based documents [59]. These systems can enable the uploading and exchange of dispensed script data [59,60] and support monitoring the dispensing of controlled medicines to minimize misuse of pharmaceuticals [60].

#### Remote Monitoring

Remote monitoring describes a range of digital technologies that can passively monitor, assess, and potentially manage patient care regardless of whether they are with a health professional [61]. They include technologies such as cardiac devices implanted into a patient on the recommendation of a health professional, but also devices such as pulse oximeters that consumers can access regardless health professional input [62].

#### Bespoke Databases

In addition to more formalized repositories of clinical information, a portion of EHD is collected by bespoke databases. This can include databases of patient information set up by individual health professionals using free electronic capture tools such as REDCap (Research Electronic Data Capture; Vanderbilt University) [63] to collect data on care quality and outcomes [64,65]. It can also include data from specialized medical devices and hardware such as bioimpedance spectroscopy machines [66,67] and surgical robots [68,69]. The type of data collected by medical hardware and devices varies depending on the machine. These data frequently include metrics on how end users are interacting with the device. As the data collection tools are often custom built, they can also often be flexible and adaptable to the data collection needs of the organization or specialty area [70].

### Consumer and Community Data Sources

#### Overview

The widespread access to digital technologies such as smartphones and internet access has created a plethora of potential sources of EHD generated by consumers and the public. Many of these data sources were not explicitly designed to collect data on the health and well-being of individuals, but researchers and other stakeholders have sought to understand their value. There is still a recognized challenge in harnessing these data, particularly integrating them with clinical data sources [71]. The data in this category are generally input into these ICTs by consumers or individuals in the community rather than the health workforce.

#### Smartphone, Web, and Desktop Applications

Smartphone, web, and desktop apps can be defined as self-contained programs optimized for these platforms. In health care, these applications are designed to improve health and well-being using different designs and functions [71]. The use of health applications is still mostly community and consumer driven, limiting the potential of these technologies to transform health care [71]. Due to the diversity of health conditions applications have been developed for, there is considerable breadth to the data they could collect. Data collection fits across four broad categories: (1) automatic collection using smartphone features such as GPS to track distance traveled and cameras to collect photo diaries; (2) linkage with Internet of Things devices that collect, record, and transmit data such as weight scales and blood pressure monitors; and (3) manual input of data by users, including recording calories, menstrual cycles, medication compliance, and blood glucose [71]. Despite the breadth of data that could be collected, researchers have noted that most users are using them to collect data on things such as fitness activities, daily activity levels, and tracking sleep [72].

#### Web-Based Patient Reviews

A diverse array of websites on the internet let consumers publish reviews and provide web-based ratings about clinical encounters with individual health professionals or health care organizations. Feedback provided via web-based patient reviews (WPRs) is unsolicited by the health professional. These data can be structured (ie, ratings) or unstructured (ie, free text comments) [73]. WPRs have been shown to influence health care consumers’ health professional selection and decision-making [74]. In addition, some research suggests that specialist clinicians have used WPR data to improve patient communication and workflows [75,76].

#### Appointment Booking Systems

Appointment booking systems allow patients to book and manage their medical appointments on the web [77]. While traditionally these systems were developed and managed by individual health care practices, they are increasingly being developed and maintained by software companies. Health care practices often embed the booking application into their website, and structured data collected within the system are often stored on the cloud [28].

#### Web-Based Communities

These data describe various sources of information on health, including social media data and data from forums and other web-based communities. Web-based communities are peer-to-peer communities where people with common interests can gather virtually to share experiences, ask questions, and provide emotional support [78]. The data collected by web-based communities are typically unstructured and text based but can vary based on the platform. Social media describes internet-based applications with a social dimension, typically by enabling users to post content and interact with the content of others almost synchronously [79]. Data collected by social media platforms are varied but include the content in individual posts, metrics on engagement with the content, and information on the network individuals choose to follow [18]. Data generated from web-based sources possess distinct characteristics compared to other web-based information, such as patient reviews. This is because the main objective of the web-based platform is not specifically to gather health data. However, there are many web-based communities for discussion of health conditions, including diabetes management [80], cancer management [81], and general health and wellness [82].

#### Web-Based Search Engines

Web-based search engine data describe information on specific terms individuals input into search engines to find results relevant to their queries. Data collected by search engines are primarily terms users input into an individual search engine. A widely used source for such information is Google Trends, which provides a database of terms frequently searched on their platform. It allows for comparing individual terms across different regions and languages [83]. This is among the earliest web-based sources of health information, with initial research using this data to monitor public health trends dating as far back as 2008 [84].

### Technological Data Sources

#### Overview

The high level of digitization in contemporary society means that individuals interact with a significant amount of ICT daily. Some of these ICTs are actively sought out by individuals, such as purchasing a smartphone or an activity tracker. Other ICTs in this category are interacted with less overtly, such as search engines. Typically, data in this category are passively collected by technology as end users interact with it rather than actively input by consumers or the health workforce.

#### Wearables and Devices

Wearable devices are digital technologies that an individual wears as an accessory and that may also use sensors to track health information [7]. The data they collect are typically structured. Although used to collect health data, wearable devices were not originally developed to support health care. They were developed by the fitness industry to track activity to support individual health and well-being [85]. Wearable devices are increasing in popularity in the community [86], translating into an increased interest in their use in health care.

#### Direct Digital Health Care

Technology is increasingly used to enable direct consumer health care models, which may entirely digitize care delivery. These systems include health check stations that enable individuals to monitor their health without interaction with a traditional health care organization [87]. In addition, there are systems that integrate artificial intelligence (AI) technologies with human expertise, serving as digital-first entry points to care. They offer AI-based triage and referral services to deliver preventative care [88-90]. Finally, large non–health organizations are also entering this space to offer end-to-end health and wellness management services, including home installation of apps and devices and dedicated teams of virtual clinicians and managers [88].

## Challenges and Limitations of the Current Digital Health Information Systems

### Quality, Completeness, and Interoperability

Ensuring data input into health information systems is complete, of high quality, and able to be linked and shared across different systems within and across organizations is a major challenge with the current digital health information systems. In practice, management software data completeness issues are common, particularly on older systems where administrative and patient records are not linked, potentially leading to loss of information when data are transferred between systems [48]. Similarly, PAS used in hospitals have been shown to be inaccurate for reporting care outcomes [51]. The data can have limitations due to varied coding systems across organizations, data quality and completeness, and other issues [52]. In EMRs and EHRs, interoperability can be a major challenge, particularly a lack of integration across care contexts such as primary and acute care [46]. Registries have also had issues with interoperability due to instances of variation in standards used to define common data elements across different registries [44].

Interoperability is also a major challenge for emerging consumer health information repositories such as smartphone apps. Many apps do not include functionality that allows users to easily export data from apps and share it with health professionals [5]. In addition, the breadth of data collected by these apps could prevent integration into clinical informatics systems [71].

Data quality issues are also a significant limitation of different repositories of web-based patient health information, though the quality issues are different from clinical information systems. WPR data may not be representative, are often skewed toward a small number of health professionals, and therefore may not be sufficient for consumers to make an informed decision about a provider [91]. There is little research to show that these data can be used to measure clinical quality for individual health care providers, and the results of research in this area show mixed [91] association [73]. Coupled with this, health care organizations and health professionals have reservations about the validity of WPR data [74] and the ability of a single bad review to reach a wide audience [92]. These concerns may limit health professionals’ uptake of these data to understand the quality of care.

Social media provides a rich source of real-time health data, although it also has many limitations. These include collecting a large amount of irrelevant data, challenges in standardizing and validating data, and potential biases in the demographics and geographic location of social media users and identifying these biases in the data [84,93]. A noted limitation of search engine data is its potential to dilute cultural differences that shape web-based search behaviors or completely exclude data sets from geographic regions where those search engines are unavailable [93]. As with smartphone apps, wearables have significant data quality issues, with noted inconsistencies in data accuracy [85,94]. Wearable devices also encounter quality and completeness issues, compounded by interfaces that may not effectively highlight these gaps [95], leaving users unaware when data are not being collected [96].

### Data Security and Privacy

Data security and privacy are a major concern with contemporary information systems and are often top of mind for health care organizations. When implementing EMRs and EHRs, ensuring data stored within and shared across organizations are governed in a way that provides private and secure data exchange can be challenging [25]. In consumer-facing systems such as appointment booking engines, data have been misappropriated for financial gain [97,98], suggesting issues to be resolved about the privacy and security of these data and the social license for its secondary use.

### Complexity of Implementation and Ongoing Maintenance

Implementing any new technology in existing workflows can be challenging, and digital health information systems are no exception. Resistance to changes in workflow can be a notable barrier to the adoption of clinical information systems [28,56]. In the context of certain types of data collection, such as patient-reported outcome measures, inadequate infrastructure to support the collection of these data [99] is a major challenge. System maintenance after implementation can also be a challenge for health information systems. Limitations associated with clinical registries include the expenses related to maintenance, which often require staff with specialized skills to operate [44], and the necessity for sustained funding to support the registry in the long term [10].

Another challenge of modern information systems such as wearable devices is a lack of guidelines or tools to inform the best practice use of remote monitoring devices in clinical care [61]. Currently, very few wearable devices are subject to regulatory standards that govern other medical equipment [86], making it challenging to know which wearables are safe to use in the context of health care. Integration of these devices and the data they produce into health care remains challenging [100], as they currently do not integrate with clinical workflows or other information systems such as EHRs [86]. This same challenge occurs with health data gathered by apps, as there are currently few pathways for use in the clinical setting by health professionals [101].

### Impact on Workloads

One of the major limitations of the current digital health information systems is their impact on the workloads of health professionals. The digitization of health data collection parallels an increased administrative load for health professionals. This issue is particularly pronounced with EHRs, as health professionals typically enter data. This has been shown to increase the workloads of health professionals and contribute to burnout [25].

### Secondary Use

While considerable time and effort is spent inputting data into contemporary digital health information systems and given the vast amount of data collected, extracting and accessing these data for secondary purposes is notably difficult. Even when data can be extracted, secondary use of data from health information systems has several limitations. In the context of EMRs and EHRs, it can be challenging to obtain longitudinal insights from data as information is captured at each time point a patient interacts with the health system, which for many individuals occurs as sporadic or occasional visits [102]. Similarly, data from consumer complaints and incident reporting have limitations because they capture individual incidents not holistic care [103]. This can make it challenging to identify a strategy for improving quality and can also be an issue for determining whether a complaint is attributed to an individual or a system issue.

Another challenge supporting the secondary use of many digital health information systems is scaffolding the information or supporting end users to interpret it in different contexts from those in which it was collected. For example, registries are often primarily designed for research purposes, and they are not intended to make data readily accessible for use by health services [37]. Patient-reported outcome measure data have a similar limitation. The data are not routinely used by many health care organizations [56]. Health professionals may not use patient-reported measures due to not feeling like they have the capacity to use them, not seeing their value [56], or a lack of knowledge regarding how to meaningfully interpret the data [99]. When using these data for quality improvement, barriers include a lack of timeliness of the data, limitations in determining the cause of a poor outcome, and implementing change based on a poor outcome [104]. It has also been noted that patient-reported outcome measures are only 1 piece of performance data that health professionals have access to, and the data may be ignored if individual providers do not see themselves as outliers in the data set [104].

### Data Ownership and Consent

As health information systems increase the amount of EHD collected and move toward better interoperability, patient consent for secondary use of data becomes an increasing concern. In the context of health data, consumers freely share about themselves, such as that on social media and web-based communities, which is another challenge related to consent exists. Although individuals choose to share this content publicly, it remains unclear if users are comfortable having their data used for research and other secondary applications [84].

Another major challenge with contemporary digital health information systems is data ownership. While all the data collected belongs to patients, the data are housed in information systems that consumers do not have access to. The value of these data is being increasingly recognized, and this is acting as a barrier to access. For example, the device manufacturer often controls access to data from remote monitoring devices, which can raise complex legal and ethical concerns [62]. These technologies collect large amounts of data that may be vulnerable to privacy breaches or be coopted for commercial purposes [105], and custodianship of these data is widely contested [18]. Data collected by bespoke databases in devices has value as a commercial asset [17,18], and vendors may not want to make it available to end users. Furthermore, the data collection architecture in these systems is not governed by any standards, so linking and comparing data, even if it can be accessed, is difficult.

## Actioning EHD in Practice

### Overview

EHD has great potential to transform many aspects of the health sector. There is a growing body of research exploring how data can be used to predict patient outcomes [64,68,106], understand care quality [51,55,107], and personalize treatments [21]. Approaches such as those described in learning health system processes can increase the actionability of the EHD to ensure it improves care processes and outcomes [24]. There are also a growing number of ICTs designed to increase the actionability of EHD from different sources. Much research has been undertaken into visualizing EHD from clinical sources such as PAS and EMRs to uncover insights for end users of the tools [66,108] and how approaches such as learning health system processes can increase the actionability of the EHD. Electronic patient portals use digital technology, such as a website or a smartphone application, to provide patients access to personal health information [109]. These platforms are often linked to EHRs to increase accessibility to data, help involve patients and caregivers in clinical decision-making, and improve communication between patients and their health care team [110,111]. Similarly, initiatives such as OpenNotes, which makes clinical notes available to patients and caregivers, improve transparency and patient-centered care [112,113].

**Figure 2:**
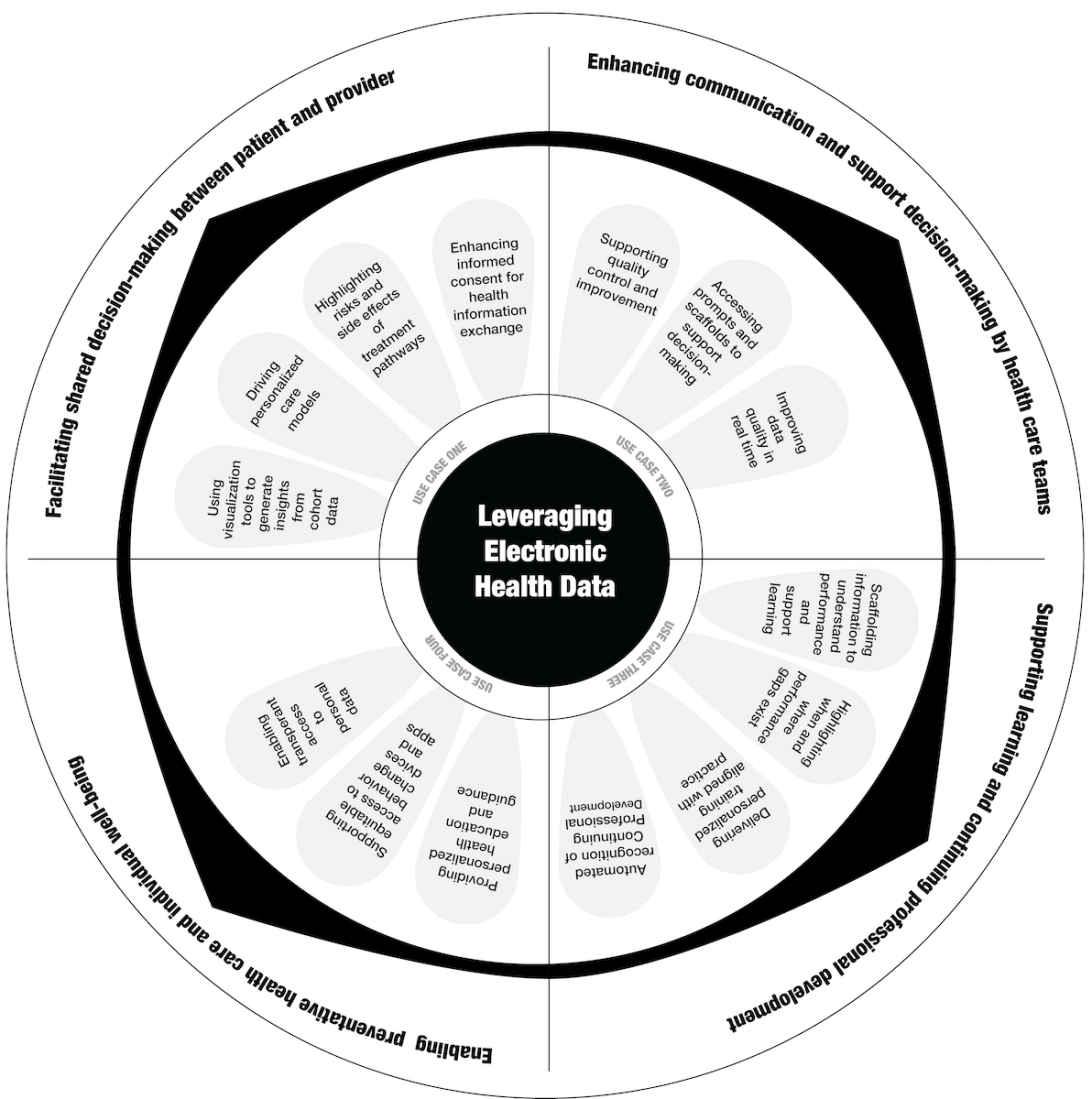
A visualization of the 4 different use cases and a high-level summary of how electronic health data can underpin it in the future.

In the following subsections, 4 use cases describe how ICT supports existing care models and how the data collected might be harnessed to transform service provision, health, and well-being. Some of these use cases describe scenarios increasingly realized in the health sector, partly due to the rapid adoption of digital platforms in response to the COVID-19 pandemic. Other use cases are informed by the research but describe hypothetical scenarios requiring evidence to be translated into practice before they can be fully realized by the health sector. These scenarios are designed to illustrate and prompt reflection on how EHD can be harnessed to generate actionable insights and transform health care in the future. Figure 2 presents a visualization of the 4 different use cases.

### Use Case 1: Informing Decision-Making in Consultations

Digital technologies and the EHD they collect can be used to strengthen patient consultations with health professionals. The value of technology to support patient interactions both within and outside of the consultation is increasingly being recognized as virtual care. Virtual care describes non–face-to-face clinical care professionally enabled through digital mechanisms [27]. In the current clinical practice, health professionals have limited access to clinical decision support tools that harness EHD and support shared decision-making [114]. Genomics data are increasingly available to health professionals to support precision medicine [57] and are likely to be more prominent in future clinical decisions. EHD from patient applications and wearable devices can be challenging for health professionals to access in consultations. When data are available, there is limited guidance on how to incorporate these data [115,116]. In addition to data access challenges, there is some evidence that the use of digital technologies during the consultation may impact patient-provider communication and rapport [117,118].

In the future, access to EHD during consultations may enable shared decision-making between patients and health professionals. This could be achieved by visualizing cohort data within an organization to help patients understand their likely outcomes for a given procedure at that specific care center [66]. It can also support more personalized decision-making in the consultation, such as by harnessing visual interfaces presenting EHD to help patients assess the risk of undergoing a specific procedure [43], supporting more informed consent processes. Finally, patients could consent to sharing health application and wearable device data with their health professional so that the data can be reviewed and discussed in the consultation to understand patient compliance with treatment plans and tailor care to align best with the patient’s lifestyle. Alongside this, the increasing application of disruptive technologies such as AI to EHD could streamline administrative processes in health care. Although not directly beneficial in the consultation, the actionability of EHD for such applications should reduce the administrative burden on health professionals and allow more time for service delivery.

### Use Case 2: Health Professional to Health Professional Interactions

Health care teams currently underuse EHD. While digital technologies such as videoconferencing have been successfully adopted as tools to support team meetings [119], the uptake of ICTs for visualizing data has been slower. There may be a need to modify physical meeting spaces to better incorporate technologies for presenting these data [120].

In the future, there will undoubtedly be greater access to EHD by individual health professionals and teams. Given the considerable time health professionals spend on data entry [25], there will likely be an increasing expectation that they will have access to these data in return for the workload burden. Access to technologies to visualize and scaffold EHD will provide health teams with a holistic view of each patient’s care journey, transforming all aspects of team interactions, including clinical decision-making, quality improvement, research, and learning. Furthermore, ready access to data by health care teams will likely have an important role in ensuring quality control as health professionals incorporate visualization tools into team meetings to review processes and outcomes of care. As part of these team reviews or as individuals, health professionals will likely use digital technologies presenting these data to review the quality of information, annotate points of interest, and edit and improve data in real time. As the application of AI in health data becomes more reliable, there will also likely be an increase in prompts and scaffolds provided by advanced analytics tools that can support decision-making within health professional teams.

### Use Case 3: Personalized Training and Reflective Practice

Health professionals regularly dedicate considerable time to engaging in education and training activities to stay up to date on the latest evidence [121,122]. Health professionals have limited access to EHD to reflect on their practice and inform decisions about professional development. Despite this, regulatory bodies expect that EHD will be used in training and professional development to maintain registration to practice [123,124]. Research suggests that health professionals would like greater access to electronic data for educational purposes [125].

In the future, health professionals, teams, and organizations will be able to leverage the plethora of EHD to understand care delivery and outcomes and enable personalized reflective practice and learning. A new range of educational technologies will be available to engage health professionals in their data to develop a rich understanding of how their behavior can change care quality. This learning approach could create authentic clinical narratives and link it with evidence-based approaches to technology enabled learning. This would focus efforts in health professional knowledge and skill development and enable accreditation bodies to recognize quality in learning experiences rather than quantity of time spent engaging in learning.

### Use Case 4: People Using Their data for Preventative Care and Health and Wellness Management

Patients have limited access to data collected by health care organizations about their treatment pathway. While technologies such as patient portals [109] and open notes [113] are making these data more available to patients and their caregivers, uptake of these solutions is limited. However, there may be an increase in patient portal adoption as regulation requires health care providers to increase the accessibility of EHD for individual patients [57]. Coupled with this, vendors from outside of health care are increasingly entering the sector to provide direct-to-consumer care by harnessing AI to enable services to be delivered when and where the consumer desires [88,89].

The health care transparency movement has also explored the value of making cost and quality information about health services available to the public [126,127], but these data are not consistently published for all organizations. There is also a plethora of consumer-centric apps and wearable devices collecting extensive data on individual activities, both general and health-specific, that enable the self-management of health and wellness [6,71,72]. Some of these innovations are beginning to be used to improve patient self-efficacy in managing chronic conditions. The increasing availability of diverse data sets also opens up exciting research opportunities. It will undoubtedly unlock new knowledge about human health and support the discovery of more personalized and adaptive approaches to maintaining health and delivering care. In the future, EHD has the potential to enable personalized preventative health and well-being solutions for members of the public. Digital technologies will increasingly make health care available outside of clinical settings, at home, in the workplace, or in whatever location suits the patient.

## Conclusions

The health sector is collecting an ever-increasing quantity of EHD. Navigating the ICTs in health care and the siloed data they collect is an ongoing challenge for stakeholders. A classification system for these data could enable stakeholders to get a high-level understanding of the complex health data ecosystem. EHD could be classified into three broad categories: (1) clinical data, (2) consumer and community data, and (3) technology-enabled data.

Understanding the complex health data ecosystem is essential if EHD is going to be leveraged to generate actionable insights that can be harnessed by ICTs to support new care models. In the last 2 years, health care has undergone considerable accelerated digital disruption as part of the COVID-19 pandemic response, which has expanded the types of EHD the sector collects. This includes a growing repository of EHD collected by new non–health corporations entering health care with direct-to-consumer products that support e-prescribing and other synchronous and asynchronous communications, which collect large volumes of health data. While these services add a rich source of information to the EHD ecosystem, they also bring challenges, including increasing the amount of data in commercial vendors’ custody.

Considerable focus to date has been placed on the value of aggregating large data sets into single repositories, which represents a significant infrastructure achievement.

However, moving forward, it is as important to understand *why* data are being collected as *how* they will be collected to ensure the correct information is available to benefit the health system and support public health and well-being.

## Abbreviations

AI: artificial intelligence
EHD: electronic health data
EHR: electronic health record
EMR: electronic medical record
ICT: information and communication technology
PAS: patient administration system
REDCap: Research Electronic Data Capture
WPR: web-based patient review
